# A drug-repositioning screen using splicing-sensitive fluorescent reporters identifies novel modulators of *VEGF-A* splicing with anti-angiogenic properties

**DOI:** 10.1038/s41389-021-00323-0

**Published:** 2021-05-03

**Authors:** Eleanor Star, Megan Stevens, Clare Gooding, Christopher W. J. Smith, Ling Li, Monica Lamici Ayine, Steve J. Harper, David O. Bates, Sebastian Oltean

**Affiliations:** 1 Institute of Biomedical & Clinical Sciences, Medical School, College of Medicine and Health, University of Exeter, St Luke’s Campus, Exeter, EX1 2LU UK; 2 Department of Biochemistry, University of Cambridge, Hopkins Building, Tennis Court Road, Cambridge, CB2 1QW UK; 3 Division of Cancer and Stem Cells, School of Medicine, University of Nottingham, Queen’s Medical Centre, West Block, D floor, Nottingham, NG7 2UH UK

**Keywords:** High-throughput screening, Drug development

## Abstract

Alternative splicing of the vascular endothelial growth factor A (*VEGF-A*) terminal exon generates two protein families with differing functions. Pro-angiogenic VEGF-A_xxx_a isoforms are produced via selection of the proximal 3′ splice site of the terminal exon. Use of an alternative distal splice site generates the anti-angiogenic VEGF-A_xxx_b proteins. A bichromatic splicing-sensitive reporter was designed to mimic VEGF-A alternative splicing and was used as a molecular tool to further investigate this alternative splicing event. Part of VEGF-A’s terminal exon and preceding intron were inserted into a minigene construct followed by the coding sequences for two fluorescent proteins. A different fluorescent protein is expressed depending on which 3′ splice site of the exon is used during splicing (dsRED denotes VEGF-A_xxx_a and EGFP denotes VEGF-A_xxx_b). The fluorescent output can be used to follow splicing decisions in vitro and in vivo. Following successful reporter validation in different cell lines and altering splicing using known modulators, a screen was performed using the LOPAC library of small molecules. Alterations to reporter splicing were measured using a fluorescent plate reader to detect dsRED and EGFP expression. Compounds of interest were further validated using flow cytometry and assessed for effects on endogenous *VEGF-A* alternative splicing at the mRNA and protein level. Ex vivo and in vitro angiogenesis assays were used to demonstrate the anti-angiogenic effect of the compounds. Furthermore, anti-angiogenic activity was investigated in a Matrigel in vivo model. To conclude, we have identified a set of compounds that have anti-angiogenic activity through modulation of *VEGF-A* terminal exon splicing.

## Introduction

Alternative splicing (AS) is one of the main levels of gene regulation in the eukaryotic cell; it affects >94% of genes in humans^1,2^. Through AS, the diversity of proteins in the cells (and therefore, protein functions) is greatly increased. There are clear studies showing that splice isoforms are not just a small modulation of the main isoform function, but rather encode functions as diverse as different genes^3^. Indeed, through AS the same gene is even able to encode opposite functions in a cell; for example, the apoptotic gene *Bcl-2* has two protein isoforms including an anti-apoptotic isoform (Bcl-xL) and a pro-apoptotic isoform (Bcl-xS), which is achieved by switching an alternative 5′ splice site in exon 2^4^.

**Fig. 1:**
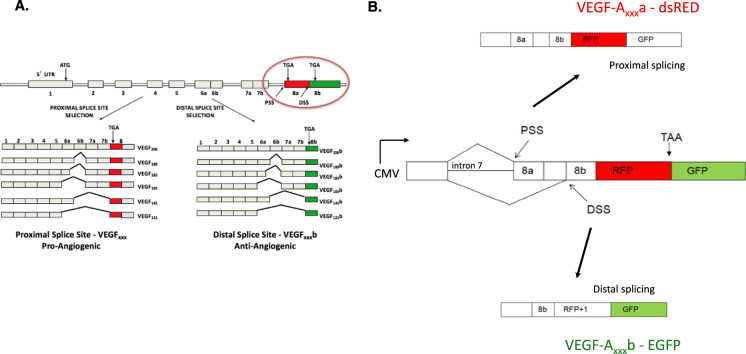
*VEGF-A* splicing-sensitive fluorescent reporter design. **A** Endogenous *VEGF-A* splicing patterns; terminal exon 8 alternative 3′ splicing results in the anti-angiogenic “b” isoforms. **B** Parts of *VEGF-A* gene (intron 7 and relevant regions of exon 8) have been inserted into the bichromatic reporter in such a way that splicing at the proximal splice site (PSS) of exon 8 results in formation of dsRED and then a stop codon is encountered, so EGFP is not formed. When the distal splice site (DSS) is used, there is a shift in the reading frame (dsRED + 1) and a fusion with EGFP is formed.

A similar example is found in vascular endothelial growth factor A (*VEGF-A*). Through usage of an alternative 3′ splice site, a novel family of isoforms (so called “b” isoforms or VEGF-A_xxx_b) is produced that have the same number of amino acids as the canonical isoforms, but differ in the sequence of the last six amino acids – see Fig. 1A^5^. Binding to VEGF receptor 2 is affected; therefore, VEGF-A_xxx_b isoforms act as antagonist/partial agonists. There has been a considerable amount of data from laboratories across the world showing that the “b” isoforms have anti-angiogenic activity both in vitro and in vivo^6–10^. The balance between the pro- and anti-angiogenic isoforms has been shown to be regulated by various signalling pathways: the canonical, proximal splice site is under the control of the splice factor SRSF1 which in turn is regulated by the splicing kinase SRPK1; the distal splice site is defined by the splice factor SRSF6 whose action is modulated by the kinase Clk1^11^. More importantly, it has been shown that they have a functional significance in disease, meaning that manipulation of splice isoform levels is able to rescue phenotypes. For example, the “b” isoforms are low in cancer and diabetic nephropathy; however, overexpression or administration of recombinant VEGF-A_165_b protein decreases tumour growth in xenografts^12^ and decreases albuminuria in diabetic nephropathy^13^. Normalisation of the VEGF-A_xxx_a/ VEGF-A_xxx_b ratio is therefore an attractive therapeutic target and makes finding small molecules that can trigger the splicing switch a worthy endeavour.

A useful tool in screening for small molecule splicing modulators is the so-called splicing-sensitive fluorescent reporter (SSFR), which is designed to give a different fluorescence outcome depending on the splicing pattern^14–16^.

We report here an unbiased, repositioning screen using a bichromatic SSFR designed to mimic the splicing ratio of VEGF-A_xxx_a to VEGF-A_xxx_b isoforms. We have found a series of molecules, called ESSOs (named after the researchers’ initials), that have been shown, upon validation, to have anti-angiogenic activity ex vivo and in vivo and to decrease tumour growth in xenografts, through a change in *VEGF-A* splicing.

## Results

### The design and validation of a splicing-sensitive fluorescent reporter to mimic *VEGF-A* terminal exon splicing

A bichromatic reporter template has been used based on previously published designs^14,17^. This was engineered to mimic *VEGF-A* exon 8 splicing. The reporter (termed pRG-VEGF8ab) consists of the endogenous *VEGF-A* intron 7 and required parts of exon 8, followed by the coding sequences for two fluorescent proteins [see details in Methods section and Stevens et al.^15^]. Depending on which 3′ splice site is used for exon 8, a different fluorescent protein is expressed. When the canonical, proximal splice site is used, dsRED is formed followed by a stop codon, so EGFP is not produced. When the distal splice site is used, there is a shift in the reading frame (dsRED + 1) and a fusion protein with EGFP is produced (Fig. 1B). Therefore, dsRED denotes the pro-angiogenic VEGF-A_xxx_a isoforms, and EGFP denotes the anti-angiogenic VEGF-A_xxx_b isoforms.

While this reporter has been used and validated by our lab in vivo in a transgenic mouse model^15^ for the purpose of this study – to use it in a screen – we wanted to pursue more validation in vitro. This is to ensure it reproduces the endogenous *VEGF-A* splicing pattern, including response to several compounds known to affect *VEGF-A* terminal exon splicing through inhibition of SRPK1^18^.

**Fig. 2:**
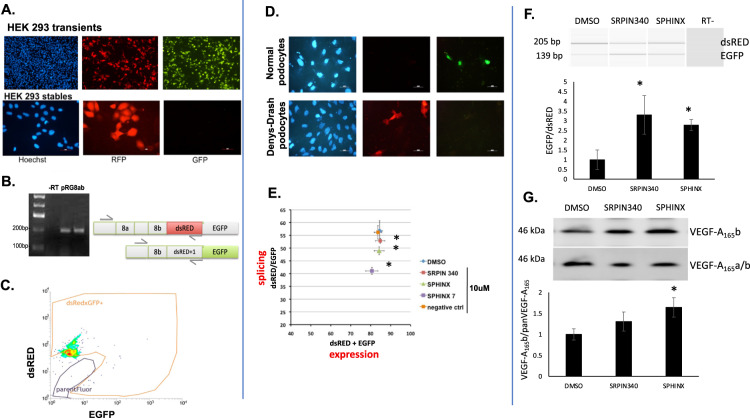
Validation of the reporter in cell culture. **A** Transfection of the reporter in HEK293 cells – upper panel: transient transfections (24 h) show both dsRED and EGFP signal due to multiple copies of the reporter; lower panel – stable transfected cells display only dsRED fluorescence corresponding to the *VEGF-A* terminal exon proximal splice site usage in these cells. **B** RT-PCR for the reporter shows correct splicing in HEK293 cells. **C** FACS analysis of HEK293 stably transfected with the reporter shows dsRED fluorescence only (parentFluor area is the trace for naïve untransfected cells). **D** In proliferating conditionally immortalised human podocytes, selection of the reporter’s distal splice site leads to EGFP expression. No dsRED, only EGFP expression was observed in transfected podocytes indicating an anti-angiogenic splicing pattern (upper panel). In Denys Drash Syndrome (DDS) podocytes, where the VEGF-A_xxx_b isoforms are not expressed, we see only dsRED expression. Transfection efficiency is estimated at 20%. **E** SRPK1 inhibition increases use of the pRG-VEGF8ab reporter distal splice site (dsRED/EGFP), as measured by the plate reader. *n* = 3, *P < 0.05. No significant difference in dsRED+EGFP measurements. **F** The reporter was further validated after stable transfection into PC3 cells, Both SRPK1 inhibitors resulted in an increase in the EGFP/dsRED ratio. *n* = 3, *P < 0.05 vs DMSO. **G** To determine whether the reporter splicing mimics that of endogenous VEGF-A, PC3 cells were treated with the same SRPK1 inhibitors. The protein VEGF-A_165_b/panVEGF-A_165_ ratio was increased after treatment with SRPIN340 and SPHINX, although only significant with the more potent SRPK1 inhibitor, SPHINX. *n* = 3, *P < 0.05 vs DMSO.

The endogenous *VEGF-A* is present exclusively as the pro-angiogenic isoform in HEK293 cells^19^. Transient transfection of pRG-VEGF8ab into HEK293 cells resulted in both a dsRED and EGFP signal being produced (Fig. 2A, upper panel). This is not surprising, as in a transient setting there are many copies of the reporter inside the cells and this can swamp the splicing machinery and the regulation of a particular splice site^20^. However, when stable transfected HEK293 cells are obtained, the reporter displays only dsRED fluorescence (Fig. 2A, lower panel), which is expected for the exclusive proximal splice site usage in this cell line. RT-PCR analysis confirmed this expected splicing pattern, with only a band observed for dsRED (Fig. 2B), and FACS analysis shows exclusively dsRED positive cells (Fig. 2C). On the other hand, when pRG-VEGF8ab is transfected into proliferating conditionally immortalised podocytes, reported before to have high levels of VEGF-A_165_b^21^, EGFP can predominantly be seen (Fig. 2D, upper panel). However, when transfected in Denys-Drash podocytes – previously reported to express predominantly the pro-angiogenic VEGF-A_165_a isoform^19^ – only dsRED is observed as expected.

To further validate that the reporter behaves as expected we have used a set of SRPK1 inhibitors. We have previously shown^18,19,22^ that SRPK1 phosphorylates the splice factor SRSF1 and consequently promotes the proximal splice site in the *VEGF-A* terminal exon, defining the pro-angiogenic VEGF-A_xxx_a isoform. If SRPK1 function is abrogated either genetically by knocked-down or using inhibitors (SRPIN340 or SPHINX), there is a switch in splicing towards the distal splice site and promotion of the anti-angiogenic VEGF-A_xxx_b isoform. We would therefore expect that treatment of cells transfected with the pRG-VEGF8ab reporter with SRPK1 inhibitors to shift fluorescence of the reporter from dsRED towards EGFP. Indeed, as seen in Fig. 2E, there is a progressive and significant decrease in dsRED/EGFP ratio (indicating splicing changes) when HEK293 cells stably transfected with pRG-VEGF8ab are treated with inhibitors with increasing potency (SPHINX7 more potent than SPHINX which is more potent than SRPIN340). Remarkably, when treated with a control inhibitor that mimics SPHINX7 but it is chemically inert, there is no shift in the splicing ratio. Additionally, there is no movement with any inhibitor in the (dsRED + EGFP) values on the X-axis, indicating that these inhibitors do not affect the reporter expression.

We next stably transfected PC3 cells with pRG-VEGF8ab to further validate the reporter at the mRNA level using RT-PCR. In control treated PC3s, dsRED was predominantly expressed at the mRNA level, which equates to this cells line having a high expression of pro-angiogenic VEGF-A isoforms^18^. Treatment of the transfected PC3 cells with SPRIN340 and SPHINX (10 μM, 48 h) resulted in a significant increase in the EGFP/dsRED ratio at the mRNA level (*P < 0.05; Fig. 2F). We next used Western blotting to determine the protein expression of the endogenous VEGF-A isoforms in PC3s exposed to the same conditions. Both SRPIN340 and SPHINX increased the VEGF-A_165_b/panVEGF-A_165_ ratio, although this was only significant with the more potent SPHINX (*P < 0.05; Fig. 2G).

Of note, even in an in vivo setting the reporter responds accurately to SRPK1 inhibitors – when transgenic mice harbouring the pRG-VEGF8ab reporter are treated intraperitoneally with SPHINX injections, there is a reporter switch in the expected direction ascertained by both fluorescence and RT-PCR^15^.

Additionally, we have recently reported^23^ that a natural blueberry extract (DIAVIT) upregulates production of VEGF-A_165_b isoforms in podocytes. Indeed, when stably transfected HEK293 cells were treated with DIAVIT, the reporter dsRED/EGFP splicing ratio decreases as expected, in a dose-dependent manner (Supplementary Fig. 1).

The fact that the reporter responds in a similar way to the endogenous *VEGF-A* terminal exon splicing gave us confidence that it can be used to study the regulation of this splicing event.

### Screening for *VEGF-A* splicing modulators

In a quest to uncover novel anti-angiogenic compounds that are able to modify *VEGF-A* splicing we set-up a screen using the splicing reporter described. We have chosen a repositioning screen using the LOPAC (library of pharmacologically active compounds; Sigma, Inc). The LOPAC library is formed of 1280 compounds that are pharmacologically active, either marketed drugs or pharmaceutically relevant structures. The library is designed to cover most signalling pathways and major drug target classes. Due to one of the goals of this project – to find modulators of angiogenesis with one major target being tumour angiogenesis – we chose the prostate cancer PC3 cell line for screening.

**Fig. 3:**
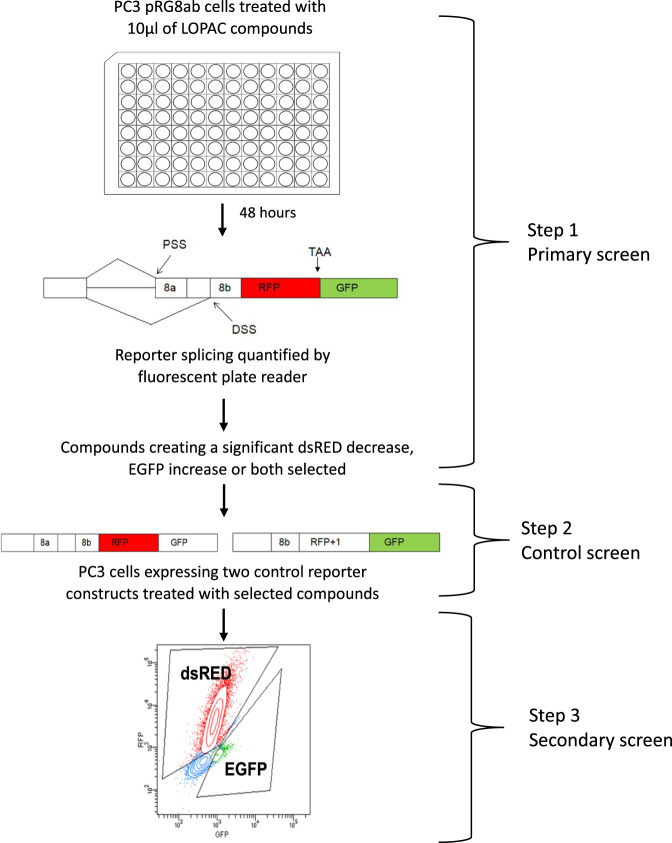
Screening the LOPAC library for small molecules that effect the alternative splicing of the *VEGF-A* reporter construct. PC3 cells transfected with the fluorescent VEGF splicing reporter were treated with the LOPAC compound library and reporter splicing measured using a fluorescent plate reader (Primary screen, step 1). Hit compounds were used to treat non-splicing control reporter constructs (Control screen, step 2). Flow cytometry was used to measure the effect of the hit compounds on reporter splicing (Secondary screen, step 3).

The screen was done in three steps (see Fig. 3). Following the primary screen, the first positive list of compounds was screened for elimination of false positives that may directly affect fluorescence or RNA stability. This was done using cells stably transfected with control reporters that lack the intron, cannot be spliced and mimic the resulting dsRED or EGFP RNA of the two spliced isoforms (see step 2 in Fig. 3). Finally, due to the inherent errors in any methodology, the resulting compound list after the control screen were re-screened using a different method – FACS. A hit-list of 9 compounds (called ESSOs) resulted after this last step of screening – see Table 1.

**Table 1:** Screened compounds shown to reduce the ratio of dsRED/EGFP fluorescence intensity on the plate reader.

Compound	Shortened name	EGFP	dsRED
Trovafloxacin mesylate	ESSO01	↑	↓
Melatonin	ESSO02	↑	↓
5-[(4-Ethylphenyl)methylene]-2-thioxo-4-thiazolidinone	ESSO03	↑	↓
N6-2-(4-Aminophenyl) ethyladenosine	ESSO04	↑	↓
8-Bromoadenosine-3′,5′-cyclophosphate sodium	ESSO05	–	↓
Flupirtine maleate	ESSO06	↑	–
RepSox	ESSO07	↑	–
GW2974	ESSO08	↑	–
4-(2-Aminoethyl) benzenesulfonyl fluoride hydrochloride	ESSO09	↑	–

### ESSO compounds validation

**Fig. 4:**
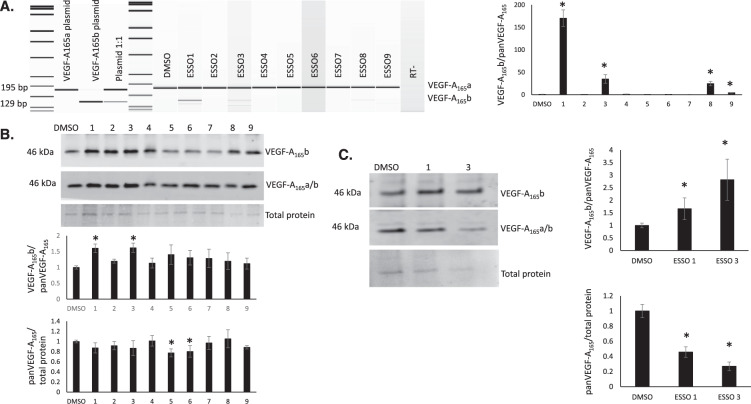
Validation of ESSO compounds in vitro. **A** The effect of ESSO compounds on the alternative splicing of endogenous VEGF-A mRNA transcripts. RNA was extracted from PC3 cells following 48 h treatment with ESSOs (10 μM), reverse transcribed and amplified by PCR. Anti-angiogenic isoform transcripts were expected to be 66 bp smaller than pro-angiogenic transcripts. ESSO1, 3, 8 and 9 resulted in a significant shift in splicing to increase VEGF-A_165_b at the mRNA level. *n* = 3, *P < 0.05 vs DMSO. **B** Changes in expression of anti-angiogenic VEGF-A protein isoforms upon treatment of PC3s with ESSO compounds (example western blot). Following treatment with each small molecule (10 μM, 48 h), protein was extracted and used for western blotting. The bands observed correspond to VEGF-A_165_b and VEGF-A_165_a/b isoform dimers (46 kDa). ESSO1 and 3 resulted in a significant increase in VEGF-A_165_b relative to panVEGF-A_165_, whereas ESSO5 and 6 resulted in a significant decrease in both VEGF-A_165_ isoforms. *n* = 5, *P < 0.05 vs DMSO. **C** Changes in expression of anti-angiogenic VEGF-A protein isoforms upon treatment of podocytes with ESSO 1 and 3 compounds (example western blot). Following treatment with each small molecule (10 μM, 48 h), protein was extracted and used for western blotting. The bands observed correspond to VEGF-A_165_b and VEGF-A_165_a/b isoform dimers (46 kDa). ESSO1 and 3 resulted in a significant increase in VEGF-A_165_b relative to panVEGF-A_165_, as well as a significant decrease in panVEGF-A_165_ expression. *n* = 8, *P < 0.05 vs DMSO.

To validate the main hit compounds described above, RT-PCR and western blot analysis were performed on PC3 cells individually treated with ESSO compounds at 10 μM for 48 h. At the mRNA level, ESSO 1, 3, 8 and 9 resulted in a significant switch in splicing towards the VEGF-A_165_b isoforms (Fig. 4A). Furthermore, both ESSO1 and 3 showed a significant increase in the VEGF-A_165_b/panVEGF-A_165_ ratio at the protein level in PC3 cells, whereas ESSO 5 and 6 resulted in a significant decrease in panVEGF-A_165_ expression Fig. 4B).

The effect of the compounds of the protein VEGF-A_165_b/panVEGF-A_165_ ratio was also assessed in podocytes, a cell line with a higher endogenous level of VEGF-A_165_b^19,21^ We found that treatment of podocytes for 48 h with 10 μM ESSO1 and ESSO3 resulted in a significant increase in the VEGF-A_165_b/panVEGF-A_165_ ratio at the protein level, as well as a significant decrease in panVEGF-A_165_ expression (Fig. 4C).

### ESSOs may not regulate *VEGF-A* splicing by direct interaction with the spliceosome or the RNA

**Fig. 5:**
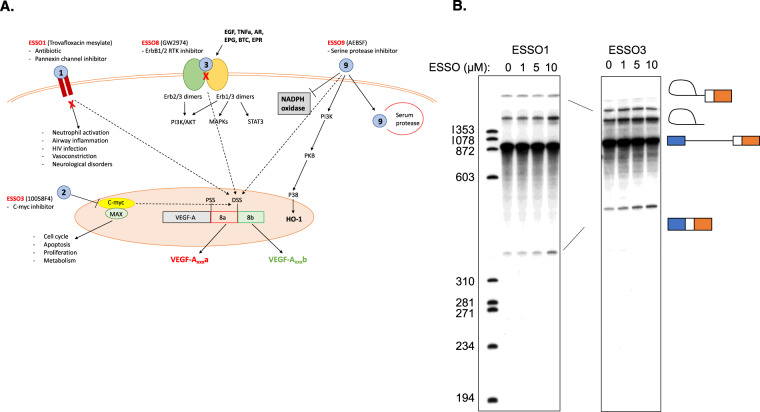
Mechanism of action of ESSOs. **A** Examples of known signalling mechanisms for ESSOs. **B** Splicing gels. Separation of products after 2 h splicing reaction. Separated on 5% denaturing polyacrylamide gels. Positions of precursor, lariat intermediate, spliced product and lariat product indicated. 5′ exon intermediate would be at 130 nt. Labelled DNA markers in left lane – sizes indicated to left.

ESSOs are part of a repositioning library composed of either substances that are approved drugs or chemicals that were developed for drug purposing. While for most of them, the mechanism of action and signalling pathways involved are known (Fig. 5A), it is not clear whether or not the new activity they are tested for (angiogenesis inhibition) occurs through the same mechanism – e.g. ESSO8 is an RTK (receptor tyrosine kinase) inhibitor – does its activity on VEGF-A splicing and angiogenesis depend on RTK signalling?

A number of small molecules are known to directly target core components of the splicing machinery^24^. To test the possibility that one or more of the ESSOs might affect *VEGF-A* alternative splicing by directly targeting components of the splicing machinery, we used cell-free in vitro splicing assays. Radiolabelled pre-mRNA substrates containing *VEGF-A* exon 8a and 8b 3′ splice sites and 365 nt of the adjacent upstream intron 7, with a constitutive *Tpm1* 5′ exon and splice site were transcribed in vitro and then incubated in HeLa cell nuclear extract. The RNAs spliced exclusively using the *VEGF-A* 8a 3′ splice site in HeLa extract (Fig. 5B and Supplementary Fig 2A). When the predicted exon 8a branch sites were weakened by A to G point mutations at −30 and −31 nt upstream (Supplementary Fig 2A), the efficiency of 8a splicing was substantially reduced, with no concomitant upregulation of 8b splicing. This suggests that splicing of exon 8b requires specific activation. We then tested the effects of ESSO compounds at 1, 5 and 10 μM concentration. The ESSOs either had no effect or modestly increased exon 8a splicing (Fig. 5B and Supp Fig. 2B). However, none of the ESSO compounds detectably activated exon 8b splicing. These results therefore suggest that the ESSO compounds do not directly target the splicing machinery to affect *VEGF-A* 8a vs 8b splicing, but more likely act upstream to regulate splicing indirectly. The assay and our conclusions have limitations though, as we do not have a positive control for exon 8b activation in this assay.

### ESSO compounds inhibit angiogenesis ex vivo

**Fig. 6:**
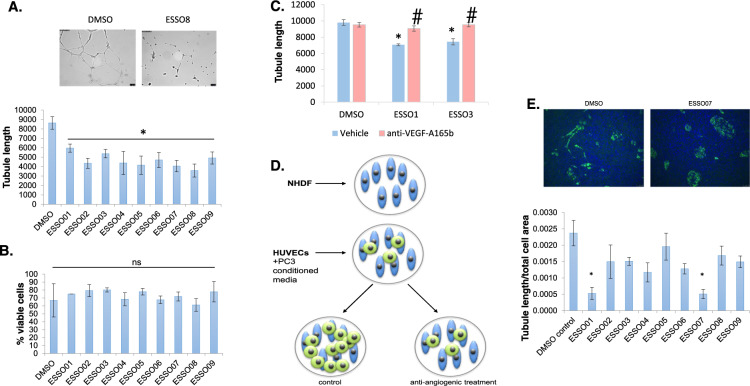
ESSOs have anti-angiogenic function ex vivo. **A** Using a Matrigel angiogenesis assay ESSOs 1-9 resulted in a statistically significant decrease in HUVEC tubule length, thus angiogenesis, compared to DMSO control. *n* = 5, *P < 0.05. **B** HUVEC cell viability is not altered by treatment with ESSO compounds compared to DMSO control. *n* = 3, *P* = ns. **C** Anti-angiogenic VEGF-A isoforms mediate ex vivo inhibition of endothelial tube formation. The presence of anti-VEGF-A_165_b (56-1; 10 μg/ml) significantly reversed the effect of ESSO 1 and 3 on tubule length. Three images of each well were captured and quantified. *n* = 3, *P < 0.05 vs. DMSO control. ^#^*P* < 0.05 vs. ESSO control. **D** Schematic of the fibroblast (NHDF) and endothelial cell (HUVEC) co-culture experiment to assess angiogenic potential in the presence of ESSO-treated PC3 conditioned media. **E** Endothelial cell tubule formation was significantly reduced by ESSOs 1 and 7 during endothelial fibroblast co-culture. Ten microscopic fields of each co-culture were quantified for the total length of endothelial tubules formed normalised to the total cell area. *n* = 3, **P < 0.01 vs DMSO.

We further wanted to test whether the ESSO compounds are able to inhibit angiogenesis. We first used them in ex vivo Matrigel assays. There was a significant decrease in the Matrigel tubule length with all ESSO compounds compared to DMSO control (Fig. 6A), suggestive of anti-angiogenic properties. This was not due to a toxic effect on the HUVECs as there was no difference in cell viability with the various treatments, as assessed by Trypan blue staining (Fig. 6B). To show that the effect is, at least in part, due to a switch in *VEGF-A* splicing when using ESSO1 and ESSO3, we performed a rescue experiment in which antibodies specific to VEGF-A_165_b (56-1; 10 µg/ml) were also added while treating with ESSO1 and 3, as examples. As expected, this partially rescued the effect of the ESSOs on Matrigel tubule length (Fig. 6C).

To further test ESSOs anti-angiogenic properties, another assay was employed; an endothelial cell - fibroblast co-culture assay (Fig. 6D)^25^. Normal human dermal fibroblasts (NHDF) were plated and after settling for 24 h, HUVECs were added on top. Conditioned media from PC3 cells treated with ESSOs (as above) was added to the co-culture. Following incubation, cells were fixed and stained with CD31 (an endothelial cell marker) and DAPI, to visualise all cells in a particular well. While in control conditions (DMSO), endothelial cells start proliferating and migrating to form contacts (Fig. 6E, left upper panel), this is inhibited in the presence of most ESSOs, of which ESSO1 and ESSO7 were significant (see Fig. 6E, right upper panel and quantification in the lower panel).

Furthermore, we found that addition to HUVEC cells of conditioned media from PC3 cells treated with either ESSO 1 or ESSO 3 resulted in a significant decrease in the phosphorylation of VEGFR2 in comparison to a DMSO control, further indicating that the increase in the VEGF-A_165_b/VEGF-A_165_a ratio in response to ESSO treatment has anti-angiogenic properties (Supplementary Fig. 3).

These results are consistent with our hypothesis that ESSOs have anti-angiogenic activity ex vivo, some through an effect on *VEGF-A* splicing.

### ESSOs inhibit angiogenesis in vivo

**Fig. 7:**
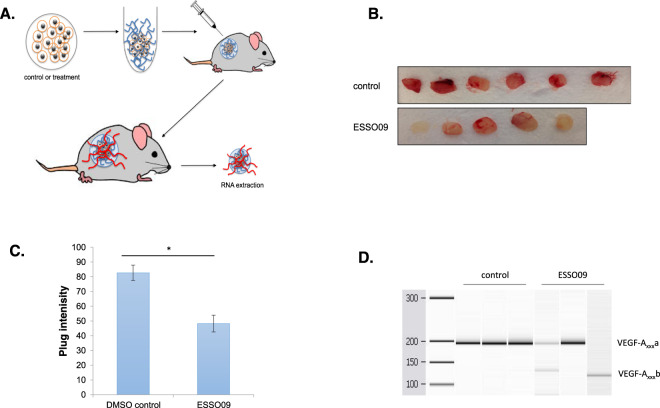
ESSO9 inhibits angiogenesis in vivo. **A** ESSO9-treated PC3 cells (48 h) were mixed with Matrigel. The Matrigel and control cells were injected subcutaneously into nude mice. Four days post-injection, the plugs were removed and RNA was extracted. **B**, **C** Photographs were taken of each plug and colour intensity quantified using ImageJ software. There was a significant reduction in ESSO9 plug intensity. *n* = 6, *P* = 0.0002. **D** RT-PCR for VEGF-A splice variants showed a switch in splicing to increase VEGF-A_xxx_b isoforms in some ESSO9-treated plugs compared to DMSO controls (*n* = 3).

To further investigate the effects of certain ESSOs on angiogenesis, we employed an in vivo assay using Matrigel plugs. We chose first to test ESSO 9 because out of the four ESSOs that showed significant switch in *VEGF-A* splicing, ESSO9 had the lowest efficiency (Fig. 4A). Cells were incubated with either DMSO or ESSO9, mixed with Matrigel and implanted subcutaneously in the back of nude mice. Normally, small vessels from the mouse vasculature grow into the plug (see schematic Fig. 7A). In the presence of anti-angiogenic activity, there is less vasculature in the plug. Indeed, this is the case of ESSO9 treatment, as there was less red colour in the ESSO9-treated plugs (Fig. 7B, C). Furthermore, we confirmed a *VEGF-A* splicing switch to increase the VEGF-A_xxx_b isoform at the mRNA level in some of the plugs treated with ESSO9 (Fig. 7D). Finally, we repeated this experiment with ESSO1 and obtained similar results (Supplementary Fig. 4). This shows that the two ESSOs, even if with different efficiencies in switching *VEGF-A* splicing, work well in vivo to inhibit angiogenesis.

## Discussion

Although discovered more than 50 years ago, with the development of novel methods to analyse genomes globally in the last 15–20 years, AS has appeared once again in the scientific limelight because of its widespread presence in human genes. AS has emerged as a central mechanism in gene regulation that is able to modify the functional repertoire of a cell through production of many diverse proteins from a single gene. With the characterisation of numerous AS isoforms came also the realisation that many diseases express specific splice isoforms or that normal ratios of isoforms are disrupted in disease. While a certain percentage of these events may be just a silent by-product of the pathological process progression, it is clear that many of them have functional significance and indeed, manipulation of their expression or splicing ratios is beneficial to the disease phenotype. There is the possibility for splicing manipulation to work in a variety of diseases, not only in diseases caused by splicing-related mutations. This has therefore opened a new area where novel therapeutics – modulators of AS – may be designed.

One strategy for modifying splicing outcomes is the use of splicing-switching oligonucleotides (SSOs). These are complementary short RNAs designed to anneal either at splice sites or to mask various regulatory sequences. While there are still hurdles to overcome until they become good drugs in terms of systemic distribution, bioavailability, etc, there are already successful examples like the FDA-approved SSOs to treat spinal muscular atrophy or Duchenne muscular dystrophy^26^.

Another strategy is the use of small molecules to switch splicing outcomes. Initially, this was not thought to be feasible, as there were questions on how specific small molecules can be for the hundreds of thousands of splice sites that exist. In the splicing field this was argued to be possible, as the three-dimensional conformation of the RNA at splice sites combined with specific binding of splice factors is able to give a unique conformation to which a small molecule should be able to bind. However, this remained under doubt until experimental data appeared – to note two studies from Genentech and Roche using big-size compounds library to find small molecules that can switch SMN gene splicing in spinal muscular atrophy. Indeed, compounds that intercalate between splice factors and RNA have been found that are able to modulate the splicing site targeted and be more specific than some of the kinase inhibitors used today in the clinic^27,28^.

Small molecules that switch splicing may act either by (i) interfering with signalling pathways (e.g., inhibiting a splice-specific kinase that phosphorylates and activates a specific splice factor); (ii) interfering with splice factor binding at the splice sites and/or regulatory sequences; (iii) disrupting protein–protein interaction needed at a specific splice site; or by iv) altering the tertiary structure of RNA at splice sites^29^. Indeed, one of these mechanisms has been described for modulation of VEGF-A_xxx_a to VEGF-A_xxx_b ratio: inhibitors of the splicing kinase SRPK1^11,19^.

The aim of the present study was to identify novel small molecules that are able to switch *VEGF-A* splicing to increase the VEGF-A_xxx_b isoforms with an unbiased, repositioning screen using a bichromatic SSFR. The screen identified nine FDA-approved compounds, ESSO1-9, which was narrowed down to four compounds (ESSO 1, 3, 8 and 9) that had the most consistent effect on splicing of the endogenous *VEGF-A* gene. These compounds showed anti-angiogenic properties both ex vivo and in vivo, which is indicated to be due to their effect on *VEGF-A* splicing, demonstrating their therapeutic potential in disease.

While understanding the complete mechanism through which each ESSO compound acts to switch *VEGF-A* splicing is beyond the scope of this manuscript, here are some details of the ESSO compounds of most interest (see also Table 1, Fig. 4A and Supplementary Fig. 5):

- *ESSO1 - trovafloxacin* – is an antibiotic from the fluoroquinolones class; it acts in bacteria by inhibiting DNA gyrase and topoisomerase IV. It has been retracted from the market due to potential hepatotoxicity; microarray analysis of hepatocytes treated with trovafloxacin has revealed widespread gene expression modification including RNA processing genes^30^. Interestingly, a screen using the same library as our study – LOPAC – has identified trovafloxacin as a potent inhibitor of pannexin channels. These channels have been involved in a broad range of functions from neutrophil activation to vasoconstriction and neurological disorders^31^. We show here that trovafloxacin significantly increased the endogenous VEGF-A_165_a/VEGF-A_165_b ratio in PC3 and podocyte cells, resulting in anti-angiogenic properties ex vivo.
- *ESSO2 – melatonin* – is a hormone secreted by the pineal gland, involved in regulation of the sleep-wake patterns. While it has been previously known that melatonin has anti-tumour and anti-angiogenesis effects, a recent study has described a novel mechanism through which melatonin controls VEGF-A_xxx_a to VEGF-A_xxx_b splicing, and consequently, the vasculature development in the sheep pituitary gland^32^. Melatonin is therefore a positive control for our screen. Although melatonin did not significantly switch endogenous VEGF-A splicing in PC3 and podocyte cells, it did display anti-angiogenic properties ex vivo.
- *ESSO3 – 5-[(4-Ethylphenyl)methylene]-2-thioxo-4-thiazolidinone* (10058-F4) – is a c-Myc inhibitor that specifically inhibits c-Myc-Max heterodimerization and function, thus preventing transactivation of c-Myc target gene expression. C-Myc is a transcription factor that has been extensively studied due to its role as a proto-oncogene as it plays an important role in the control of proliferation, apoptosis and differentiation; its aberrant expression is seen in multiple human cancers, including acute myeloid leukaemia and pancreatic cancer. There are many reports linking c-Myc to angiogenesis^33,34^. Furthermore, c-myc is reported to regulate the transcription of several splice factors including HNRNPA1, Sam68 and HNRNPH^35–37^. Inhibition of c-Myc-Max using 10058-F4 (ESSO3) has been previously reported to have a clinical benefit in some cancers through effects on growth arrest, proliferation and chemosensitivity^38,39^. However, the link to VEGF-A splicing is a novel mechanistic connection related to angiogenesis. We show that 10058-F4 significantly increased the endogenous VEGF-A_165_a/VEGF-A_165_b ratio in PC3 and podocyte cells, resulting in anti-angiogenic properties ex vivo.
- *ESSO8 – GW2974* – is a potent and selective dual EGFR and ErbB-2 receptor tyrosine kinase inhibitor. These receptors have been previously implicated in angiogenesis directly or indirectly and their aberrant expression is considered to be associated with tumour malignancy and poor patient prognosis^40^. GW2974 has been reported to inhibit tumour progression; in PC3 cells, GW2974 was found to inhibit cell growth, indicating its potential in the treatment of prostate cancer^41^. However, there are no reports linking GW2974 to the regulation of AS. Our data provides a new mechanistic link on how inhibition of these receptor relates to splicing and angiogenesis as GW2974 was found to significantly increase the endogenous VEGF-A_165_a/VEGF-A_165_b ratio in PC3 cells, as well as showing anti-angiogenic properties ex vivo.
- *ESSO9 - 4-(2-Aminoethyl) benzenesulfonyl fluoride hydrochloride (AEBSF)* – is an irreversible serine protease inhibitor. Data on the effects of AESBF on angiogenesis are very limited; however, one report has linked AEBSF to the inhibition of angiogenesis through inhibition of NADPH oxidase in vivo^42^. Our data may provide further downstream links to VEGF-A in modulating angiogenesis through the effects of AEBSF on the VEGF-A_165_a/VEGF-A_165_b ratio in PC3 cells, in addition to the anti-angiogenic properties of AEBSF both ex vivo and in vivo.

When testing compounds in repositioning screens, the mechanism for the novel effect could be completely different from the canonical one. We therefore tested whether some ESSO compounds may have a direct effect on the splioceosome/RNA by using them in a nuclear extract splicing asay (Fig. 5B). While the conclusions we can draw from this assay are limited because we do not have a positive control, we believe their effect to be through canonical signalling pathways.

A limitation of this reporter system is that the dsRED/EGFP ratio may not represent the exact splicing ratio of the endogenous VEGF-A splice isoforms at the protein level. Potential reason for this may be the stability of the mRNA or post-transcriptional regulation mechanisms. However, this does not negate the value of this dual reporter system in detecting directional changes in VEGF-A splicing.

In conclusion, we believe AS, similar to other levels of gene regulation including transcription or miRs, is an area where therapeutic ideas may be developed in a range of diseases. As demonstrated with the example of *VEGF-A* AS presented in this paper, it is possible to revert disease phenotypes by modulation of AS and it is feasible to use small molecules to do this.

## Materials and methods

### Cell culture

HEK293, PC3, Podocyte, HUVEC and NHDF cells were sub-cultured from existing cultures within the lab. HEK293, PC3 and NHDF cells were cultured in DMEM (D6429, Sigma - St Louis, Missouri, USA) supplemented with 10% foetal bovine serum (10270, GIBCO – Waltham, Massachusetts, USA) and 1% penicillin streptomycin (GIBCO). Podocytes and Human umbilical vein endothelial cells (HUVECs) were cultured in EBM-2 media (Lonza – Basel, Switzerland)) supplemented with EGM-2 Bulletkit (Lonza). All cell lines were cultured at 37 °C and in a humidified incubator with 5% CO_2_.

### Reporter construction

Details about reporter design are given in Stevens et al.^15^ where we used it to construct a transgenic mouse.

### LOPAC primary screen

A library containing 1280 FDA-approved chemicals, LOPAC, Library of Pharmaceutically Active Compounds (LO4200, Sigma-Aldrich) was used. Chemicals in the library were dissolved DMSO at a stock concentration of 10 mM. PC3 pRG8ab cells were trypsinised and diluted to 8 × 10^4^ cell/ml using DMEM. In all, 100 μl of the cell solution was seeded into each well of a black 96-well plate. The cells in each 96-well plate were treated with DMSO only and 16 different compounds from the LOPAC in triplicate at 10 μM. Wells of the outside edges of 96-well plates were not used for treatments or measured for fluorescence. Plates were incubated for 48 h at 37 °C. Changes in reporter alternative splicing caused by treatment with LOPAC compounds were measured using a VICTOR X multi-label plate reader (Perkin Elmer – Waltham, Massachusetts, USA). Each plate was measured using VICTOR plate reader three times. DMSO treated wells were used as control measurements and statistically compared to treated wells from the same 96-well plate using a one-way ANOVA.

### FACS, RT-PCR and western blotting

These were performed by standard methods/conditions – for details please see Supplementary Information

### Endothelial cell tube formation assay

This was performed as previously described;^23^ for details please see Supplementary Information

### Angiogenesis co-culture assay and CD31 Immunofluorescence

These were performed as previously described;^19^ for details please see Supplementary Information

### Trypan Blue cell viability assay

Cells were washed and trypsinised and described previously and diluted to an approximate concentration of 2 × 10^5^ cells per ml. In all, 0.5 ml of cell suspension was transferred to a screw cap tube and mixed thoroughly with 100 μl of 0.4% Trypan Blue stain. Cells and the stain were incubated for 5 mins at room temperature. A haemocytometer was filled and used to count cells under a microscope. Stained cells were non-viable and unstained cells were viable.

### Matrigel plug angiogenesis assay

PC3 cells were cultured in media containing 10 μM ESSO or DMSO for 48 h. Cells were detached from culture plates and diluted in DMEM to a concentration of 2 × 10^7^ per ml and placed on ice. Matrigel basement membrane protein (734–1100, VWR) was thawed on ice. In total, 100 μl of cell suspension was mixed with 400 μl of Matrigel and 10 μM of the test compound. The mix of cells and matrix was subcutaneously injected into each upper flank of Crl:CD1-*Foxn1*^*nu*^ nude mice (males, 2 months old; Charles River). Four days post-injection, mice were culled by cervical dislocation (Schedule 1) and the Matrigel plugs were extracted. Images of each plug were taken, and their colour quantified using Photoshop as an indication of blood vessel infiltration into the matrix. Plugs were flash frozen in liquid nitrogen. In total, 1 ml of Trizol was added to each plug and samples were homogenised for RNA extraction as described.

### In vitro splicing assays

were carried out in HeLa cell nuclear extract as described previously^43^. Transcripts contained 365 or 165 nt of *VEGF-A* intron 7 immediately upstream of the exon 8a 3′ splice site and 220 nt of exon 8, including the 8b 3′ splice site followed by a BamHI restriction site. The 5′ exon, 5′ splice site and first 150 nt of the intron were from rat *Tpm1* exon 1. Run-off transcripts were generated with T7 RNA polymerase from BamHI linearised template in the presence of α-[^32^P]UTP. Spliced RNA products were separated on 5 or 6% denaturing polyacrylamide gels and analysed by autoradiography or phosphorimager for quantitation.

### Statistical analysis

Comparisons of two datasets were performed using Students’ *t* test or a Mann–Whitney *U* test, depending on whether the data met the normal distribution. A comparison of three or more groups was performed using one-way analysis of variance (ANOVA) with Dunnett’s post-test. *P* < 0.05 was considered to indicate a statistically significant difference.

## Supplementary information

Supplementary Figures

Supplementary methods
