# Supplementary material for: A drug-repositioning screen using splicing-sensitive fluorescent reporters identifies novel modulators of VEGF-A splicing with anti-angiogenic properties
Source: Oncogenesis. 2021 May 3;10(5):36. doi: 10.1038/s41389-021-00323-0 (PMC8093282; doi:10.1038/s41389-021-00323-0)
Supplement: Supplementary file 1 — Supplementary Figures [file 41389_2021_323_MOESM1_ESM.pptx]

## Slide 1
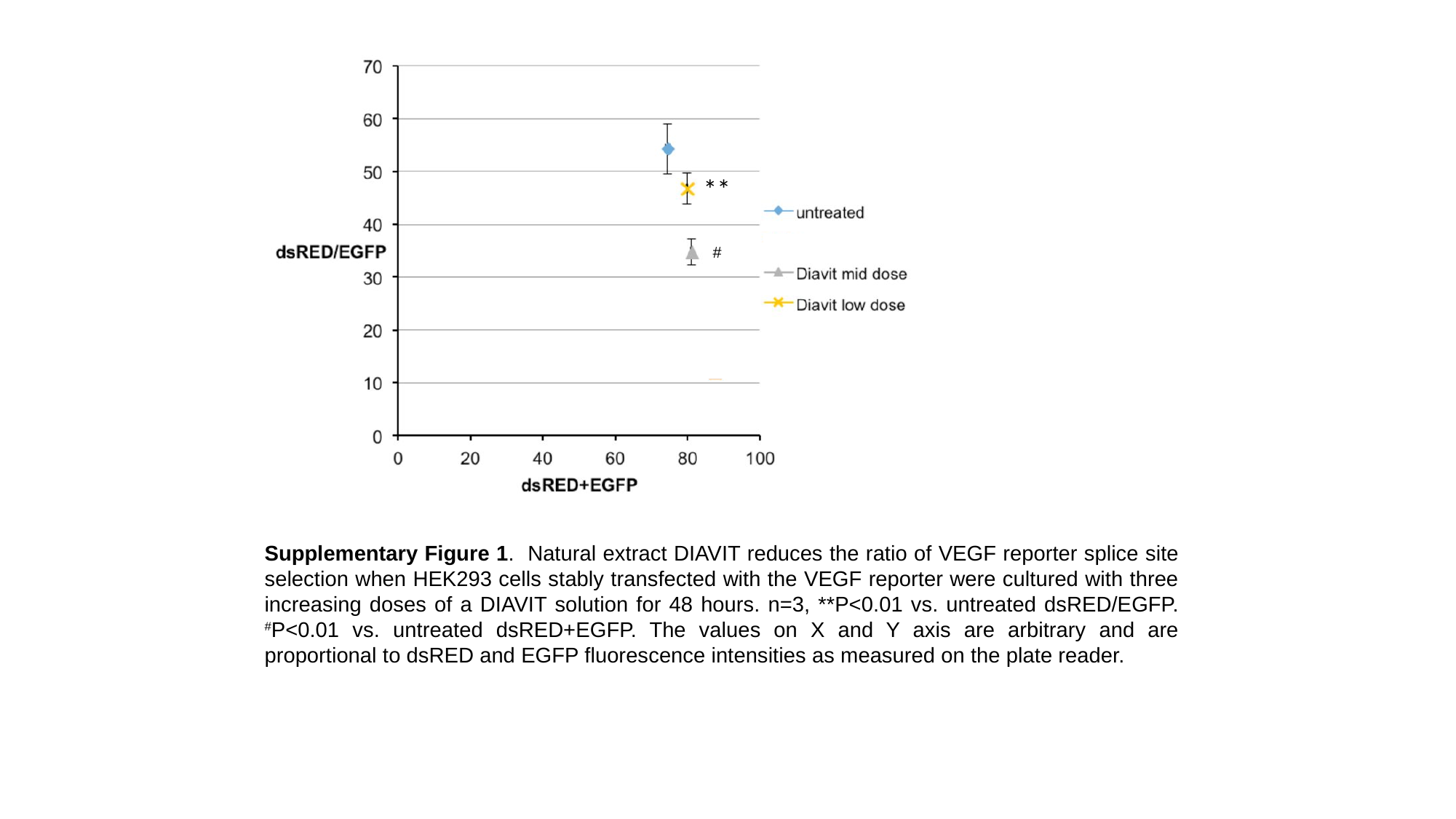

**
#
Supplementary Figure 1. Natural extract DIAVIT reduces the ratio of VEGF reporter splice site selection when HEK293 cells stably transfected with the VEGF reporter were cultured with three increasing doses of a DIAVIT solution for 48 hours. n=3, **P<0.01 vs. untreated dsRED/EGFP. #P<0.01 vs. untreated dsRED+EGFP. The values on X and Y axis are arbitrary and are proportional to dsRED and EGFP fluorescence intensities as measured on the plate reader.

## Slide 2
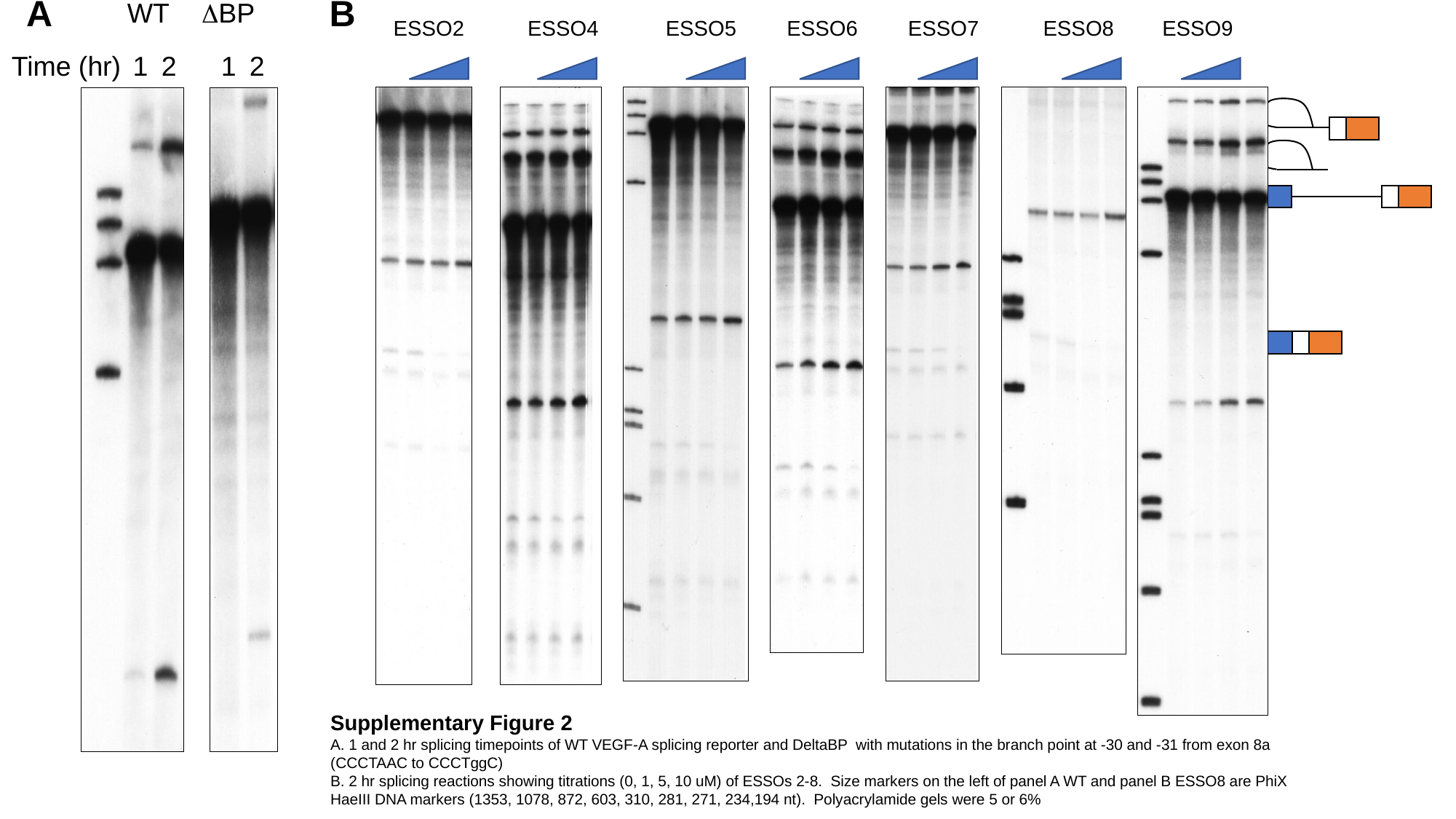

A
B
WT
DBP
ESSO2
ESSO4
ESSO5
ESSO6
ESSO7
ESSO8
ESSO9
Time (hr)
1
2
1
2
Supplementary Figure 2
A. 1 and 2 hr splicing timepoints of WT VEGF-A splicing reporter and DeltaBP with mutations in the branch point at -30 and -31 from exon 8a (CCCTAAC to CCCTggC)
B. 2 hr splicing reactions showing titrations (0, 1, 5, 10 uM) of ESSOs 2-8. Size markers on the left of panel A WT and panel B ESSO8 are PhiX HaeIII DNA markers (1353, 1078, 872, 603, 310, 281, 271, 234,194 nt). Polyacrylamide gels were 5 or 6%

## Slide 3
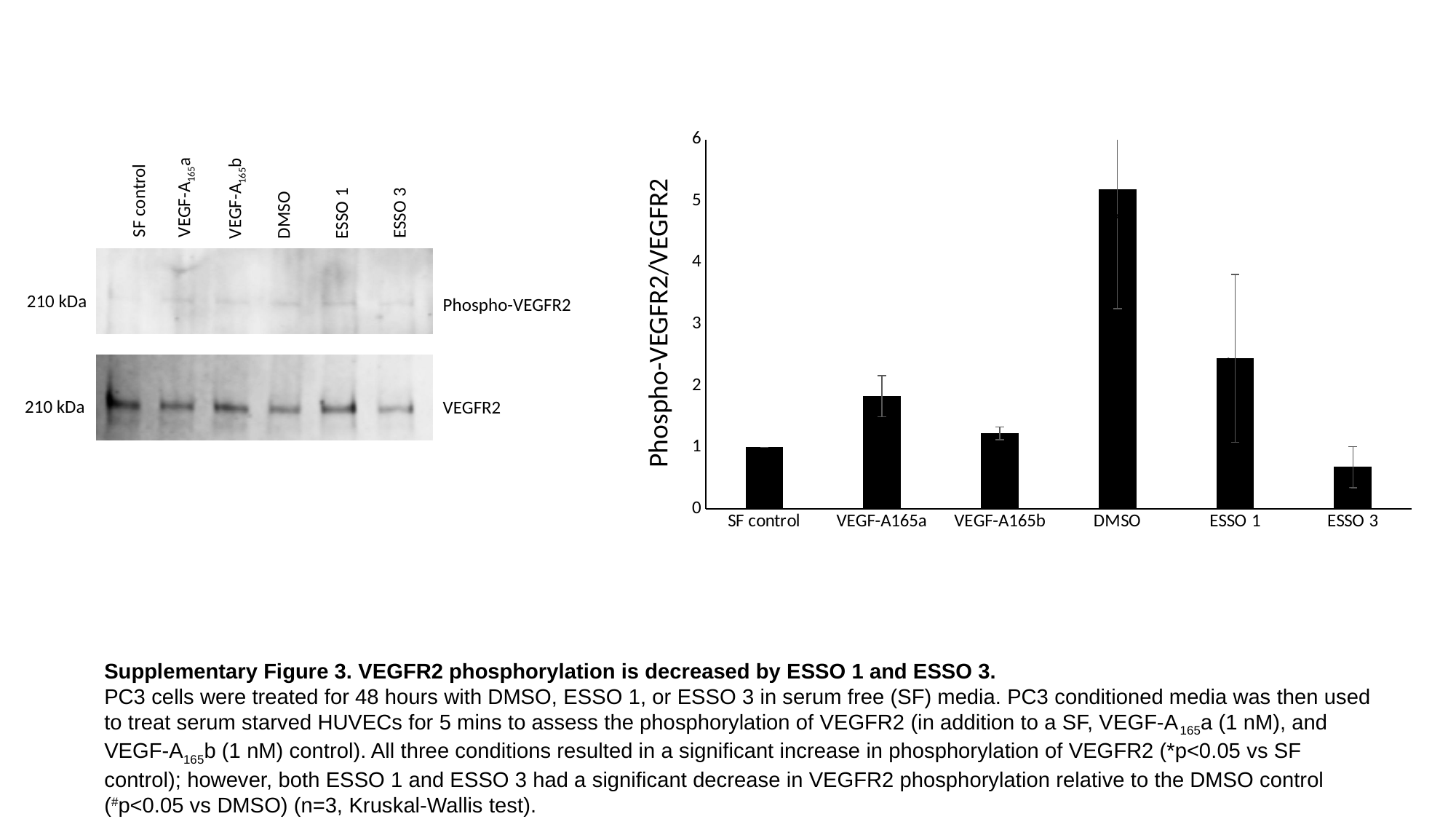

### Chart
| Category | |
|---|---|
| SF control | 1.0 |
| VEGF-A165a | 1.829164838379406 |
| VEGF-A165b | 1.2257946423582122 |
| DMSO | 5.182390118490146 |
| ESSO 1 | 2.444712574635298 |
| ESSO 3 | 0.675567084294856 |VEGF-A165a
SF control
ESSO 3
ESSO 1
DMSO
VEGF-A165b
*
Phospho-VEGFR2/VEGFR2
210 kDa
Phospho-VEGFR2
*#
210 kDa
VEGFR2
*
#
Supplementary Figure 3. VEGFR2 phosphorylation is decreased by ESSO 1 and ESSO 3.
PC3 cells were treated for 48 hours with DMSO, ESSO 1, or ESSO 3 in serum free (SF) media. PC3 conditioned media was then used to treat serum starved HUVECs for 5 mins to assess the phosphorylation of VEGFR2 (in addition to a SF, VEGF-A165a (1 nM), and VEGF-A165b (1 nM) control). All three conditions resulted in a significant increase in phosphorylation of VEGFR2 (*p<0.05 vs SF control); however, both ESSO 1 and ESSO 3 had a significant decrease in VEGFR2 phosphorylation relative to the DMSO control (#p<0.05 vs DMSO) (n=3, Kruskal-Wallis test).

## Slide 4
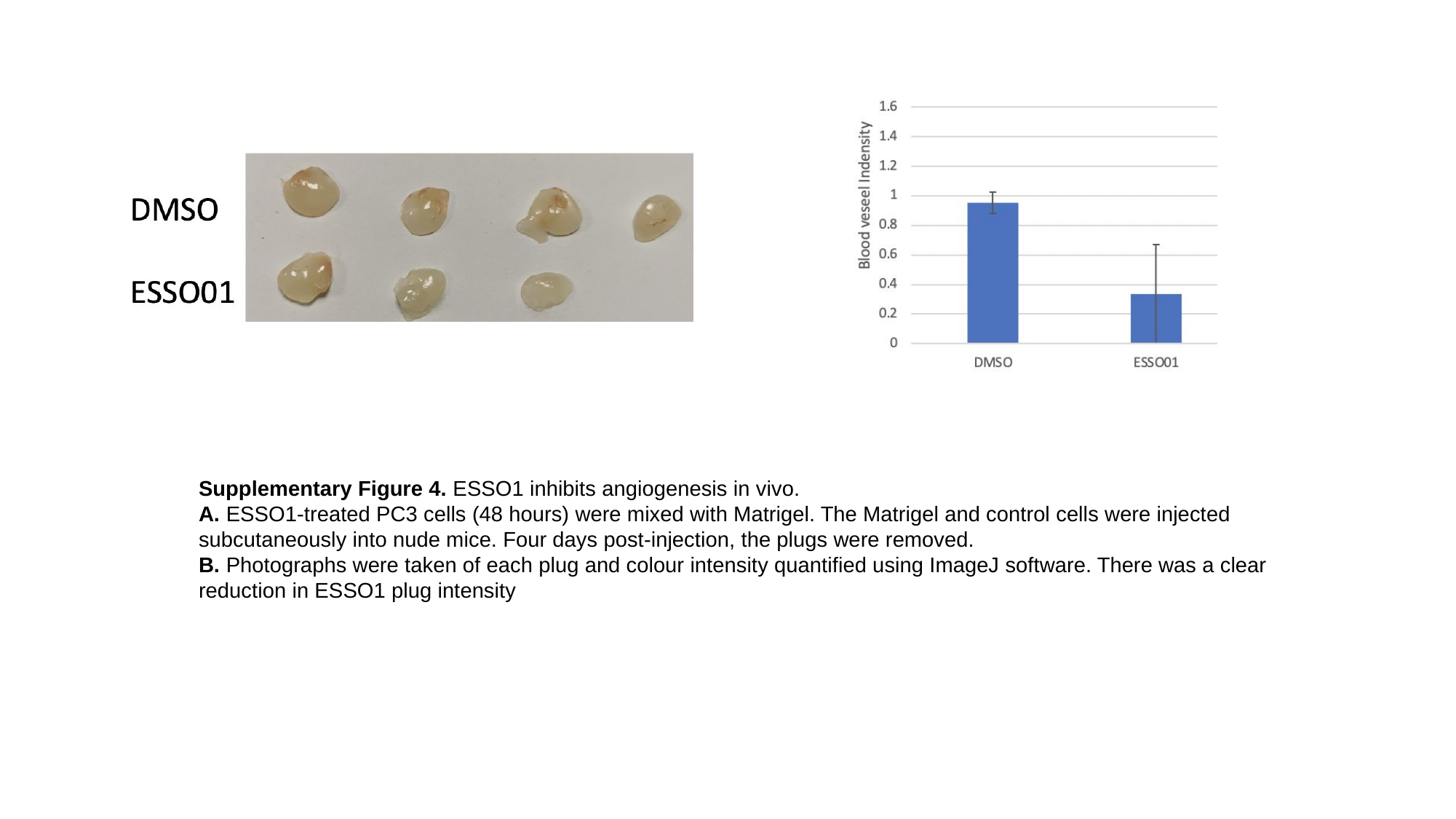

Supplementary Figure 4. ESSO1 inhibits angiogenesis in vivo.
A. ESSO1-treated PC3 cells (48 hours) were mixed with Matrigel. The Matrigel and control cells were injected subcutaneously into nude mice. Four days post-injection, the plugs were removed.
B. Photographs were taken of each plug and colour intensity quantified using ImageJ software. There was a clear reduction in ESSO1 plug intensity

## Slide 5
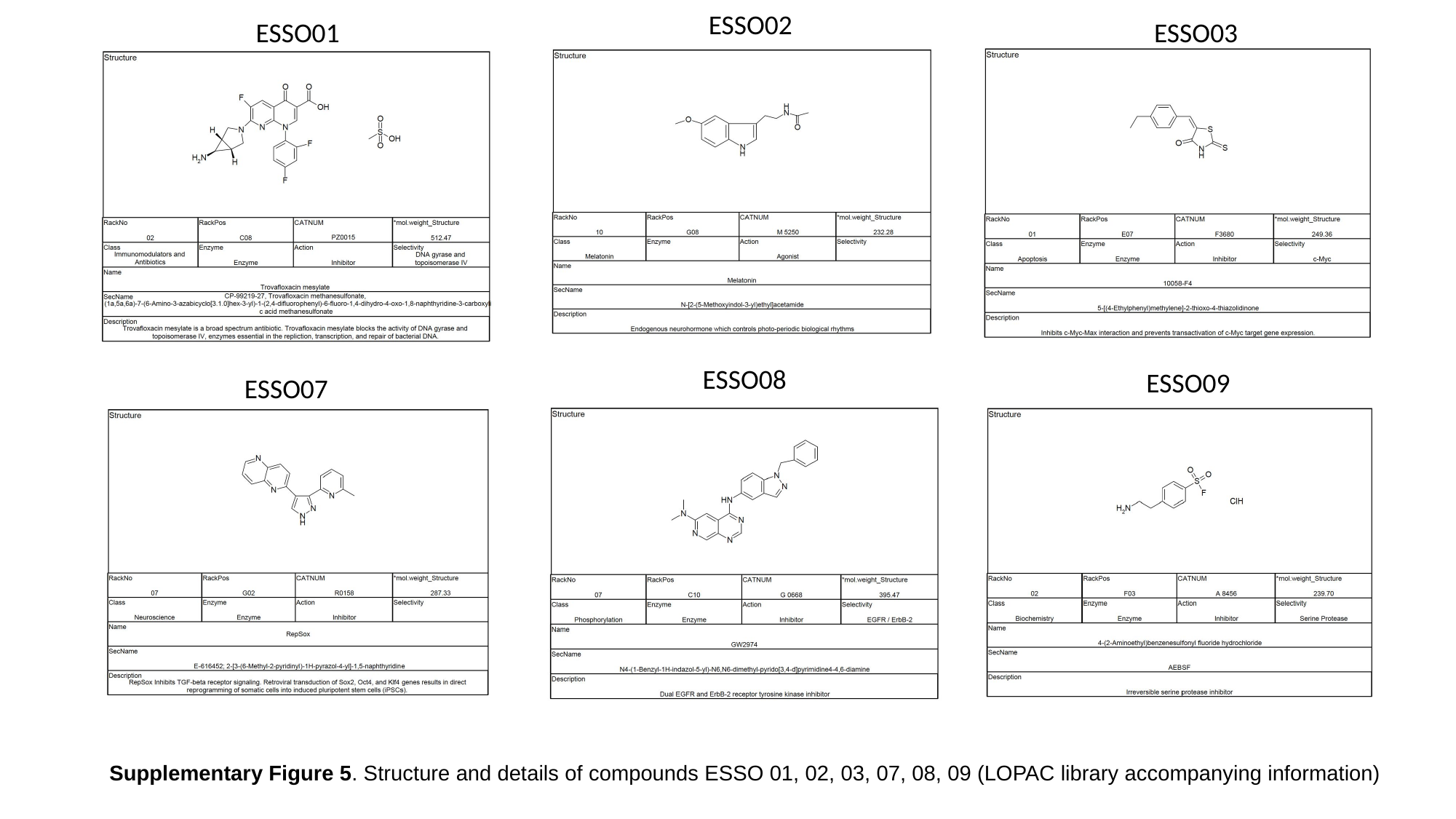

ESSO02
ESSO01
ESSO03
ESSO08
ESSO09
ESSO07
Supplementary Figure 5. Structure and details of compounds ESSO 01, 02, 03, 07, 08, 09 (LOPAC library accompanying information)
