# Supplementary material for: A drug-repositioning screen using splicing-sensitive fluorescent reporters identifies novel modulators of VEGF-A splicing with anti-angiogenic properties
Source: Oncogenesis. 2021 May 3;10(5):36. doi: 10.1038/s41389-021-00323-0 (PMC8093282; doi:10.1038/s41389-021-00323-0)
Supplement: Supplementary file 2 — Supplementary methods [file 41389_2021_323_MOESM2_ESM.docx]

**Supplementary Material**

**FACS**

HEK293 cells stably transfected with the reporter were treated with SRPK1 inhibitors at 10µM for 48 hours. After treatment, live cells were collected, and flow cytometry used to measure the percentage of cells expressing dsRED and EGFP. Percentages were used to calculate the ratio of splice site selection (dsRED/EGFP). A decrease in the ratio suggests increased selection of the distal splice site. Changes in overall reporter expression are indicated by dsRED+EGFP. The effect of treatment on alternative splicing are measured by dsRED/EGFP. FACS was performed at the Bristol University Flow Cytometry facility.

**RT-PCR**

RNA was extracted from cells using the RNeasy kit (Qiagen), and from tumours using the phenol-chloroform, before reverse transcribing to cDNA. RT-PCR was performed for pRG-VEGF8ab and VEGF-A splice variants, and qRT-PCR was performed for VEGF receptor 2.

**Western blotting**

Protein was extracted from cells using RIPA lysis buffer with protease inhibitors. Denatured protein samples were run on mini-PROTEAN® TGX Stain Free™ pre-cast gels (4-15%, BIORAD), which allow for visualisation and accurate analysis of the total protein loaded for each sample using a Gel-Doc™ EZ (BIO-RAD) imaging system. The use of this system means a housekeeping protein loading control is not required as the amount of protein on the membrane for each sample can be quantified. Once protein had been transferred on to a PVDF membrane, total protein could be quantified. Membranes were blocked in 3% BSA in TBS plus 0.3% Tween before being probed with either anti-VEGF A20 (Santa Cruz) or anti-VEGF-A_­165_b (R&D Systems) at 1:1000 dilution in 3% BSA-TBS-Tween (0.3%), at 4°C overnight. After washing membranes in TBS-Tween (0.3%), fluorescent secondary antibodies were diluted in 3% BSA-TBS-Tween (0.3%), 1:10,000. Membranes were incubated for 2 hours before washing. VEGF-A isoform expression was imaged in a Licor Clx imager. VEGF-A_165_b expression was normalised to panVEGF-A_165_ expression, and panVEGF-A_165_ expression was normalised to the loaded protein for each sample, as quantified by the Gel-Doc™ EZ imaging system (BIO-RAD). Experiments were repeated on at least three biological repeats, with the relative controls run on the same blot.

**Angiogenesis co-culture assay**

PC3 cells were cultured in T75 culture flasks. When 70-80% confluent, the media was removed and replaced with EBM-2 endothelial cell media containing ESSO compounds or DMSO control. After 48 hours, the conditioned media was removed from the PC3s and stored at -20°C.

An 11-day protocol was performed with endothelial cells co-cultured with confluent fibroblasts. On day 1, normal human dermal fibroblasts (NHDF) were cultured in DMEM supplemented with 10% FBS. Confluent NHDF cells were harvested with trypsin and counted using a haemocytometer. 3x10^4^ cells were seeded onto sterile coverslips in 12-well plates and incubated at 37°C. On day 4, media was replaced with fresh EBM-2. On day 5, 3x10^4^ HUVECs in fresh EBM-2 media were added to each well. EBM-2 media was refreshed on day 7. On day 9, the media was replaced with the conditioned media taken from PC3s treated with ESSOs. Cells were fixed and stained for endothelial markers via immunofluorescence on day 11.

**CD31 Immunofluorescence**

Media was removed from the cells and coverslips were washed with 1 ml 1x PBS before fixing for 15 mins in 1 ml 4% paraformaldehyde. After fixation, coverslips were washed twice with 1x PBS. Non-specific binding of antibodies was blocked by incubating coverslips in 3% bovine serum albumin (BSA) in 1x PBS overnight at 4°C. The co-culture was stained for the endothelial cell marker CD31 (PECAM). Monoclonal CD31 primary antibody (3528, Cell Signalling Technology) was diluted 1:500 in 1% BSA and incubated on the coverslips for 1 hour. Two washes in 1x PBS were performed before staining with Alexa Fluor 488 rabbit anti-mouse antibody (1:1500 in 1% BSA 1x PBS) for 1 hour. The co-cultures were washed two final times with 1x PBS and mounted onto microscope slides with Vectashield mounting medium containing DAPI (H-1200, Vector Labs) to visualise the nuclei.

**Endothelial cell tube formation assay**

PC3 cells were cultured in EBM-2 media and treated with ESSOs (10 μM). Following 48 hours of treatment, the conditioned media was removed and stored at -20°C. HUVECs at passage 6 or lower were grown in 6-well plates until ~70% confluent. HUVECs were cultured in EBM-2 cell culture medium including the serum and growth factors of the EGM-2 bullet kit with the exception of VEGF. Cells were treated with ESSO compounds (10 μM) or controls for 48 hours before the assay.

50 μl of Matrigel basement membrane matrix (734-1100, VWR) was seeded into the appropriate number of wells of a chilled 96-well plate. The plate was placed at 37°C for >30 minutes to solidify the Matrigel. Pre-treated HUVECs in 6-well plates were detached from culture plates using trypsin-EDTA and counted using a haemocytometer. The cells were diluted in each of the conditioned media taken from compound-treated PC3 cells and 10,000 treated cells were seeded into wells of the 96-well plate onto the solidified Matrigel. The HUVECs were incubated at 37°C for 8 hours. Treatment with ESSO compounds (10 μM) and controls was continued during this period, with/without the addition of anti-VEGF-A_165_b (56/1; 10 ug/ml). Cells were imaged using phase contrast at a magnification of 10x. Five images were captured per well. Tubule length and number of branch points were quantified for each image using ImageJ software.

**Animal experiments**

All experiments and procedures were approved by the UK Home office in accordance with the Animals (Scientific Procedures) Act 1986. Mice were maintained at the Biological Services Unit, University of Exeter, UK.

The sample size was determined by power calculations using existing data from similar experiments performed routinely in Dr Oltean’s lab. More specifically, the sample size was obtained to be able to see a significant difference (p>0.05) for tumour growth or metastatic spread with a power value of 0.80 (>80%). We have used statistical principles and formulas available on the following websites:

[www.nc3rs.org.uk](http://www.nc3rs.org.uk)

<http://www.statisticalsolutions.net/pss_calc.php>.

We have not done randomization in the animal experiments. Also no blinding of the investigator.
